# Peripartum Depression Pharmacotherapies Targeting GABA–Glutamate Neurotransmission

**DOI:** 10.3390/jcm14176177

**Published:** 2025-09-01

**Authors:** Alan C. Courtes, Louisa Smitherman, Lokesh Shahani, Jair C. Soares, Laura Goetzl, Rodrigo Machado-Vieira

**Affiliations:** 1 Department of Psychiatry and Behavioral Sciences, University of Texas Health Science Center, Houston, TX 77030, USA; 2 McGovern Medical School, University of Texas Health Science Center, Houston, TX 77030, USA; 3 Department of Obstetrics, Gynecology and Reproductive Sciences, University of Texas Health Science Center, Houston, TX 77030, USA

**Keywords:** peripartum depression, postpartum depression, GABA, glutamate, pharmacotherapies, psychopharmacology

## Abstract

Peripartum depression (PPD) represents a significant public health concern, affecting 10–17% of women globally. Traditional monoaminergic treatments demonstrate limited efficacy and delayed onset of action. The glutamate–GABA imbalance hypothesis provides a novel theoretical framework for understanding depression pathophysiology and developing targeted therapeutic interventions. This review examines emerging pharmacotherapeutic approaches targeting glutamatergic and GABAergic neurotransmitter systems for PPD treatment. Search criteria targeted randomized clinical trials investigating GABA-A-positive allosteric modulators (brexanolone, zuranolone, and ganaxolone) and NMDA receptor antagonists (ketamine and esketamine) in PPD patients. Brexanolone was the first neurosteroid to receive FDA approval for PPD, while zuranolone also shows promise. Ketamine and esketamine are also associated with reduced PPD risk, particularly with perioperative administration during cesarean delivery, though benefits are predominantly short-term. These glutamate–GABA pathway modulators represent novel therapeutic alternatives with rapid onset profiles. Further investigation and research are needed to optimize dosing protocols and patient selection criteria and to establish long-term efficacy before PPD treatment guidelines can be drafted.

## 1. Introduction

### 1.1. Epidemiology

Peripartum depression (PPD) represents a significant public health concern, affecting women during pregnancy and the postpartum period. The prevalence of PPD ranges from 10% to 15%, depending on the employed screening methods and the studied population [1]. A complex interplay is seen between socioeconomic factors, healthcare access, and cultural considerations in the manifestation and recognition of PPD. The DSM-5 does not recognize PPD as a distinct diagnosis but acknowledges the condition, providing the specifier for major depressive disorder (MDD) with peripartum onset (during pregnancy and up to 4 weeks after delivery). While PPD diagnosis remains identical to depressive disorders occurring outside the peripartum timeframe and is viewed as a heterogeneous condition, it is reasonable to consider that distinct clinical subtypes might exist, depending on factors like biological/hormonal, psychological, and social influences [2,3].

Recent systematic research has provided valuable insights into the prevalence and global distribution of this condition. A comprehensive meta-analysis examining 58 studies including 37,294 healthy mothers without prior psychiatric history found an overall postpartum depression prevalence of 17% [4]. The incidence rate was determined to be 12%, indicating that a substantial proportion of women develop depression for the first time during the postpartum period. This finding is particularly significant, as it demonstrates that even mothers without previous mental health issues remain vulnerable to PPD.

Associated suicidality risks underscore the severity of PPD. A study found that while suicide deaths and attempts are generally lower during pregnancy and the postpartum period compared to the general female population, self-harm ideation remains concerning, ranging from 5 to 14% during these periods [5]. Critically, when maternal deaths do occur, suicides account for up to 20% of postpartum deaths, and suicide represents one of the leading causes of death among women with postpartum depression [5].

### 1.2. Detection, Screening, and Risk Factors

The prompt detection of PPD is critical due to the potential adverse outcomes for both mother and fetus if PPD remains undiagnosed. These adverse outcomes include possible maternal and fetal morbidity and mortality, in addition to poor postnatal outcomes in the newborn, such as failure to thrive, developmental delays, and attachment disorders [6]. The U.S. Preventive Services Task Force (USPSTF) and the American College of Obstetricians and Gynecologists (ACOG) recommend depression screening in all expectant mothers [6]. The ACOG specifically recommends screening at the initial prenatal visit, further along in the pregnancy, and at postpartum visits, with the first visit no later than 12 weeks after birth. Screening is effective via one-step and two-step strategies that employ validated tools [7].

A one-step screening strategy utilizes the Edinburgh Postnatal Depression Scale (EPDS), a self-administered 10-item report that includes questions on mood, depression, anxiety, panic, sleep changes, guilt, and thoughts of self-harm. A four-point Likert scale, from 0 (symptoms absent) to 3 (symptoms severe), is used to score each item [8]. The EPDS remains only a screening tool and is not diagnostic; a higher score warrants additional clinical evaluation. The EPDS is advantageous due to its relative ease and rapid completion time; however, disadvantages include awkward wording and potential for scoring errors [9].

A two-step screening strategy is useful to distinguish between transient and long-lasting depression [10]. Under this strategy, patients are screened using the Patient Health Questionnaire-2 (PHQ-2) or the National Institute for Health and Care Excellence (NICE) questionnaire from the United Kingdom. Both questionnaires consist of two items that ask about depressive symptoms within the past month. This first screening step seeks to separate patients with fleeting symptoms from patients who require further screening in order to decrease unessential referrals and avoid in-depth evaluations of patients not likely to have depression [10].

There are several possible risk factors that contribute to PPD, and those risk factors can be divided in different categories, as important studies have highlighted, like sociodemographic, psychological, biological, obstetric, pediatric, and cultural risk factors [11,12]. A prior history of depression, young maternal age, tobacco use in pregnancy, prior abuse, low family socioeconomic status, and limited social support are among the most important according to a very large meta-analysis where multiple studies with populations from different countries were included [13]. Another large umbrella review highlighted factors like premenstrual syndrome, violent experiences, and unintended pregnancy as the most reliable risk factors associated with PPD [14]. Since the etiology of PPD is still unclear, most important factors, like biological, social, genetic, and psychological factors, might contribute individually, but in practice, none of those will affect diagnosis and treatment [7].

### 1.3. Clinical and Biological Correlation with MDD

The DSM-IV classified the diagnosis of major depressive disorder (MDD) by limiting onset to after delivery but within 4 weeks post birth. In contrast, the ICD-10 includes any diagnoses made within 6 weeks after birth [15]. The DSM-V maintained the above-mentioned specifier but now includes episodes that begin in pregnancy and uses the “with peripartum onset” classifier [16]. This broadens the definition of peripartum depression to include MDD diagnosed during pregnancy or up to four weeks after birth. Therefore, for the purposes of this manuscript, we have chosen the nomenclature of “peripartum depression (PPD)”, as it aligns with the new diagnostic code and with the scope of the most recent publications. As a form of MDD, PPD can meet criteria of the SIGECAPS mnemonic used to diagnose non-peripartum MDD, including sleep changes, decreased interest, guilt, decreased energy, decreased concentration, appetite changes, psychomotor changes, and suicidality [7].

As previously mentioned, history of MDD is a major predictor of future PPD [17,18,19]. There is an argument that most PPD cases are a natural recurrence of a depressive episode but, this time, coinciding with the peripartum period [20]. However, pregnancy itself may play an important role, with hormonal fluctuations triggering increased risk in vulnerable women. Similarly, the menstrual cycle, or the perimenopausal period, may be a subset, contributing to the higher prevalence of MDD [21,22,23].

Polygenic score analyses have revealed that multiple genetic factors contribute to PPD prediction, including genes related to circadian rhythms, inflammation, and psychiatric diagnoses. Specifically, in patients with MDD, the top contributing polygenic risk score for PPD was monocyte count, while in bipolar disorder patients, it was chronotype, with polygenic risk scores for inflammation and psychiatric diagnoses significantly contributing to both groups [24]. Further evidence obtained using functional magnetic resonance imaging (fMRI) supports the differentiation of PDD and MDD. Patients with PDD under resting-state fMRI exhibit hypoactivity in the amygdala and hippocampus, while non-peripartum MDD patients show hypoactivity in lateral areas related to cognition and hyperactivity in medial affective and subcortical limbic areas [25]. The same study found that when viewing emotionally happy facial expressions, non-peripartum MDD patients exhibit hypoactivity in the amygdala. Further information is needed to investigate additional linkages—or the lack thereof—between MDD and PDD.

## 2. Discussion

### Glutamate and GABA Abnormalities in Depression

Traditional monoaminergic theories of depression have given way to a new paradigm that emphasizes dysfunction in the brain’s primary excitatory–inhibitory neurotransmitter balance. The commonly known monoamine theory for MDD relies on deficiencies in monoamine neurotransmitters, such as serotonin (5-HT), dopamine (DA), and norepinephrine (NE) and synaptic gaps as the root cause of clinical depression [26]. Many antidepressants have been developed according to the monoamine hypothesis and have been commonly used worldwide.

The most prescribed class of antidepressants is represented by selective serotonin reuptake inhibitors (SSRIs), which have been proven to successfully treat clinical depression [27]. But the current monoamine hypothesis presents problems—primarily, that it fails to explain why antidepressants have a latency of response, as if antidepressants work based on the monoamine hypothesis, they should result in fast and effective action [28]. However, monoaminergic antidepressants generally take weeks to realize therapeutic effects on depressive symptoms, and up to 30% of patients with MDD are refractory [29]. In response to these limitations, depression research has branched out to encompass other areas, such as synaptic plasticity, neurogenesis, and structural brain remodeling as factors that influence mood and behavior [30].

The glutamate–GABA imbalance hypothesis represents a paradigm shift from traditional approaches, proposing that dysfunction in the delicate balance between glutamatergic excitation and GABAergic inhibition constitutes a primary mediator of psychiatric pathology [31]. This integrated framework is supported by extensive clinical and preclinical evidence demonstrating that depression involves coordinated disruptions in both neurotransmitter systems.

One pivotal study argues that this integrated approach is warranted, given that glutamate serves as the major excitatory neurotransmitter in the nervous system, with glutamatergic neurons and synapses vastly outnumbering all other neurotransmitters [31]. Astrocytes synthesize glutamine from glutamate via glutamine synthetase, which is then transported to neurons where phosphate-activated glutaminase converts it back to glutamate, serving as a precursor for both excitatory glutamate and inhibitory GABA neurotransmitters [32].

The GABAergic deficit hypothesis proposes that diverse defects in GABAergic neural inhibition causally contribute to common phenotypes of MDD and, conversely, that the efficacy of antidepressant therapies depends on their ability to restore GABAergic neurotransmission, which, in turn, helps regulate glutamatergic activity [33]. This hypothesis proposes that impaired γ-aminobutyric acid (GABA) neurotransmission plays a central and causal role in the etiology of depressive disorders by failing to adequately regulate excitatory glutamatergic transmission [34]. In depression, this delicate excitatory–inhibitory balance becomes severely disrupted through multiple interconnected mechanisms. Researchers have demonstrated that glutamatergic excess contributes significantly to cellular damage and neuronal death in brain regions critical for mood regulation while, simultaneously, GABAergic deficits fail to provide adequate inhibitory control [35].

Preclinical evidence strongly supports the causal role of coordinated glutamate-GABA dysfunction in depression [36]. Animal models demonstrate that genetic alterations affecting GABAergic function, such as GABAA receptor knockout mice, exhibit anxiety- and depression-related behaviors, cognitive deficits, and chronic hypothalamic–pituitary–adrenal axis activation, while also showing alterations in glutamatergic transmission [33].

Clinical evidence supporting this integrated dysfunction comes from multiple neuroimaging modalities. An important comprehensive meta-analysis of magnetic resonance spectroscopy studies revealed a significant reduction in absolute concentrations of Glx (a composite measure of glutamate and glutamine) in the prefrontal cortex of depressed patients [37]. Importantly, while the combined Glx signal was reduced, glutamate measurements in isolation did not differ from healthy controls. The authors of that study proposed that this observation suggests a “possible modulatory role of astrocytes in the pathophysiology of depression”. This finding further points to glial dysfunction affecting both glutamate recycling and GABA synthesis pathways.

Complementing these glutamate findings, one clinical study consistently revealed GABA concentrations in the brains of depressed patients, particularly in the occipital cortex and anterior cingulate cortex, as measured by proton magnetic resonance spectroscopy [38]. Additionally, positron emission tomography imaging has shown reduced GABAAR expression in limbic regions, including the parahippocampal temporal gyrus, indicating that both excitatory and inhibitory systems are compromised in the same brain regions critical for mood regulation [34,39]. Meta-analysis of structural neuroimaging studies also supports the involvement of both glutamatergic and GABAergic circuits in depression, demonstrating pronounced volume reductions in brain regions heavily populated by both glutamatergic neurons and GABAergic interneurons, including the anterior cingulate cortex, orbitofrontal cortex, prefrontal cortex, and hippocampus in patients with MDD [40].

Simultaneously, patients with depression exhibit reduced GABA levels in plasma, the cerebral cortex, and cerebrospinal fluid, along with altered GABAA receptor subunit composition and reduced levels of neuroactive steroids [41]. The same study showed that the administration of GABAA receptor agonists or positive allosteric modulators prevents and reverses behavioral models of depression while simultaneously normalizing glutamatergic activity.

Neuroactive steroid levels in the brain are increased during pregnancy, with a sudden decrease immediately before delivery. Interestingly, neuroactive steroids—more specifically, allopregnanolone—are also causally related to changes in the expression/function of specific GABA-A receptor subunits in the hippocampus [42]. Other studies have found that GABA levels are inversely correlated with depression scores in women at risk of developing PPD [43]. New pharmacotherapies targeting GABAergic signaling for antidepressant treatment largely purport to rely on the actions of neurosteroids on specific subtypes of GABA_A_Rs—specifically, those incorporating the δ subunit [44].

Rather than viewing glutamatergic and GABAergic dysfunction as separate phenomena, this integrated framework recognizes that depression involves a coordinated breakdown of the brain’s primary excitatory–inhibitory balance. This perspective offers a more comprehensive understanding of depression neurobiology and provides a unified rationale for developing therapeutic interventions that restore proper excitatory–inhibitory balance rather than targeting individual neurotransmitter systems in isolation.

## 3. GABAergic Pharmacotherapies (Table 1)

### 3.1. Brexanolone

Brexanolone represents a paradigmatic advancement in the treatment of postpartum depression (PPD), distinguished as the first FDA-approved therapeutic intervention specifically developed for this indication. This neuroactive steroid functions through a novel mechanism as a positive allosteric modulator of both synaptic and extrasynaptic γ-aminobutyric acid (GABA-A) receptors, diverging from conventional monoaminergic approaches.

Brexanolone demonstrates robust efficacy data across multiple controlled investigations. The initial phase 2 randomized, controlled trial established preliminary evidence of therapeutic benefit, with brexanolone achieving a clinically significant reduction of 21.0 points in Hamilton Rating Scale for Depression (HAM-D) scores, compared to 8.8 points observed with a placebo (*p* = 0.0075), following completion of a 60 h infusion protocol [45]. These preliminary findings provided the foundation for subsequent confirmatory studies. Two pivotal phase 3 trials conducted by independent research groups added compelling evidence for brexanolone’s therapeutic efficacy [46]. In the first investigation, participants administered brexanolone at 90 μg/kg/h demonstrated significant reductions in HAM-D scores (−17.7 points vs. −14.0 points for placebo, *p* = 0.0252). A total of 138 patients were randomly assigned to receive either 90 mg (*n* = 45), 60 mg (*n* = 47), or placebo (*n* = 46) in the first study. The second study, which specifically enrolled women presenting with moderate depression severity, similarly demonstrated statistically significant improvements (−14.6 points vs. −12.1 points, *p* = 0.0160). In the second study, 108 patients were randomly assigned to receive 90 mg (*n* = 54) or placebo (*n* = 54). Despite the difference in the score reduction between the studies, the findings show significant clinical reduction in depressive symptoms for both women with moderate depression and those with more severe cases. In both studies, the patients were followed for 30 days.

A pooled analysis of the three placebo-controlled trials reveals additional insights, notably demonstrating rapid onset of therapeutic action within 60 h and sustained clinical benefits through day 30 for the majority of study participants [47]. Importantly, this analysis highlighted critical safety considerations, with approximately 4% of patients experiencing serious sedation-related adverse events requiring clinical intervention. Furthermore, a critical evaluation of implementation challenges underscored the practical limitations associated with the requirement for continuous 60 h inpatient infusion and mandatory participation in a Risk Evaluation and Mitigation Strategy (REMS) program [48]. This assessment also identified important gaps in the evidence base regarding long-term efficacy and safety in specific populations, particularly women who are breastfeeding.

The safety profile across clinical trials consistently includes sedation, dizziness, and somnolence as the most frequently reported adverse events. While the requirement for inpatient administration and REMS compliance presents substantial logistical challenges, these protocols ensure appropriate safety monitoring and risk mitigation. Although several studies have demonstrated clinical benefits in the span of 30 days, these improvements were not uniformly maintained across all trial populations, indicating the need for additional research to characterize long-term treatment outcomes. Despite these implementation considerations, brexanolone’s rapid onset of action and novel neurobiological mechanism provide an important therapeutic option for women with PPD, particularly those requiring urgent symptom resolution. Future investigations should prioritize optimization of patient selection criteria, evaluation of repeat administration protocols, and comprehensive assessment of outcomes in populations inadequately represented in existing clinical trials, in addition to identifying the possible role of brexanolone in subsequent multi-modal pharmacological treatment for PPD.

### 3.2. Zuranolone

Zuranolone represents an innovative therapeutic approach for MDD through the same mechanistic pathway described for brexanolone. This neuroactive steroid has demonstrated clinical efficacy across multiple controlled investigations examining its utility as both a monotherapy and adjunctive treatment.

In initial phase 2 investigations, zuranolone administered at 30 mg daily for 14 days demonstrated statistically significant improvement in depressive symptoms compared to placebo, with a mean reduction of −17.4 points versus −10.3 points in HAM-D scores at day 15 (*p* < 0.001) [49]. The safety profile was characterized by headache, dizziness, nausea, and somnolence as the most frequently reported adverse events, with no serious adverse events documented. Subsequently, a randomized, double-blind, placebo-controlled trial conducted in Japanese adults with MDD (*n* = 250) demonstrated that once-daily zuranolone (20 mg and 30 mg) administered for 14 days resulted in significant improvements in HAMD-17 scores versus placebo (*p* < 0.05). The primary endpoint analysis revealed adjusted mean changes of −8.14 (20 mg) and −8.31 (30 mg) compared to −6.22 (placebo) at day 15. Treatment-emergent adverse events were primarily characterized by somnolence (20.7%) and dizziness (9.8%). The compound’s rapid onset of action represents a potentially significant advancement in MDD therapeutic approaches [50].

The subsequent phase 3 investigations evaluated an oral, once-daily, 14-day treatment regimen for both MDD and PPD [51,52]. The scientific rationale for this therapeutic approach derives from preclinical studies demonstrating the compound’s ability to upregulate GABA-A receptor expression and enhance inhibitory GABAergic signaling [53].

Clinical investigations have established zuranolone’s efficacy across multiple randomized, controlled trials conducted by the same research consortium. In the WATERFALL study examining MDD treatment, 50 mg zuranolone demonstrated significant improvements in depressive symptoms by day 15 compared to placebo [52]. The MOUNTAIN study, while failing to achieve its primary endpoint, which was a change from the HAMD-17 baseline on day 15, still demonstrated rapid onset of therapeutic action, with measurable improvements observed as early as day 3. This investigation also characterized the drug’s safety profile, which was determined to be favorable, with the majority of adverse events classified as mild to moderate in severity. The most commonly reported side effects included somnolence, dizziness, and sedation [51].

The long-term safety and efficacy of zuranolone were further characterized in the phase 3 SHORELINE study, which evaluated both 30 mg and 50 mg doses across repeat treatment courses extending up to one year; even after the 28-day duration of the study participants, continued to be monitored for a total of 52 weeks [54]. Among patients demonstrating initial treatment response, 42.9% in the 30 mg cohort and 54.8% in the 50 mg cohort required only a single 14-day treatment course during the study observation period. The majority of treatment-emergent adverse events were characterized as mild to moderate in severity. Most recently, the CORAL study investigated standard antidepressant therapy with or without the addition 50 mg zuranolone, demonstrating rapid improvement in depressive symptoms by day 3 compared to placebo plus antidepressant (least squares mean change: −8.9 vs. −7.0, *p* = 0.0004) [55]. The safety profile remained consistent with previously reported findings.

A recent integrated analysis of multiple clinical trials demonstrated that zuranolone improves both functional outcomes and well-being across various domains, as measured by the SF-36, with clinical benefits persisting following the completion of the 14-day treatment course and clinical improvement shown up to day 42 [56].

These investigations collectively suggest that zuranolone may represent a novel therapeutic option for MDD, characterized by rapid onset of action and the potential for episodic rather than continuous treatment protocols. Its distinctive mechanism of action targeting GABAergic signaling represents a fundamental departure from traditional monoaminergic antidepressants. The development of zuranolone represents a potential paradigm shift in depression treatment, offering a short-course therapeutic option with rapid onset of action that contrasts markedly with traditional antidepressants requiring weeks to months for full therapeutic effect. However, additional research is warranted to comprehensively characterize the long-term efficacy and optimal clinical positioning of this novel therapeutic approach.

### 3.3. Ganaxolone

Ganaxolone, a synthetic analog of allopregnanolone, functions as a potent positive allosteric modulator of GABA-A receptors with approximately 10-fold greater potency compared to benzodiazepines, as reported by two independent research groups [57,58].

Clinical investigations have evaluated the therapeutic potential of this compound for the treatment of various manifestations of depressive disorders. In a phase 2 double-blind, placebo-controlled study examining severe PPD, intravenous ganaxolone demonstrated preliminary evidence of rapid and sustained antidepressant effects. At the optimal dose of 140 mg/kg/hr, patients experienced clinically meaningful reductions in HAM-D17 scores that were sustained through day 34, with a favorable safety profile predominantly characterized by mild sedation and dizziness [57].

Furthermore, an independent research group conducted an open-label pilot investigation examining oral ganaxolone as augmentation therapy in postmenopausal women with persistent MDD, despite adequate antidepressant treatment [58]. The 8-week treatment protocol (225–450 mg BID) resulted in statistically significant improvements in depression metrics, with 44% of subjects achieving both response and remission criteria based on Montgomery–Asberg Depression Rating Scale (MADRS) scores. Notable improvements were observed across multiple domains, including sleep quality, anxiety, and irritability. While all study participants experienced sleepiness and fatigue, the antidepressant effects demonstrated durability throughout the 2-week taper period and at 3-month follow-up assessment. The findings from these investigations suggest ganaxolone’s promise as a novel therapeutic agent for depression, particularly within hormonal contexts such as PPD and postmenopausal depression. However, larger randomized, controlled trials are required for definitive determination of clinical efficacy and optimal patient selection criteria.

**Table 1:** Studies on GABAergic compounds for the treatment of peripartum depression.

Drug	Mechanism of Action	Study	Design	Phase	*n*	Key Findings	Side Effects
**BREXANOLONE**	GABA-A neuroactive steroid/neurosteroid-positive allosteric modulator (allopregnanolone analog)	Kanes et al., 2017 [45]	RDBPCT—parallel group	II	10	Mean total HAMD score changes from baseline to hour 60 and our 84. Total HAMD scores for all patients were ≤7 at all assessments points from hour 24 onward.	Sedation, acute loss of consciousness, flushed skin/face, dry mouth, and vertigo
		Meltzer-Brody et al., 2018 [46] (NCT02942004)	RDBPCT—3-arm	III	45	Reduction in total HAMD score from baseline after 60 h in both groups (60 mg and 90 mg) when compared with the placebo group	
		Meltzer-Brody et al., 2018 [46] (NCT02942017)	RDBPCT—2-arm	III	54	Reduction in total HAMD score from baseline after 60 h in both groups (60 mg and 90 mg) when compared with the placebo group	
		Epperson et al., 2023 [47]	Post hoc analysis of 3 RCTs mentioned above	Pooled analysis	102	HAMD-17 score reduction at hour 60 in patients receiving 90 mg compared with placebo	
**ZURANOLONE**	GABA-A neuroactive steroid/neurosteroid Positive allosteric modulator (allopregnanolone analog)	Deligiannidis et al., 2023 [53]	RDBPCT	III	196	Statistically significant improvement in depressive symptoms using the HAMD score at day 15 and significant improvement in depressive symptoms reported on days 3, 28, and 45	Headache, dizziness, nausea, and somnolence
		Clayton et al., 2024 [56]	Integrated data from four RDBPCTs	Pooled analysis	1003	Significant changes from baseline for 6 domains of HAMD on day 15 and changes from baseline for all 8 domains of the HAMD on day 42	
**GANAXOLONE**	GABA-A neuroactive steroid/neurosteroid-positive allosteric modulator (allopregnanolone analog)	Gutierrez-Esteinou et al., 2019 [57]	RDBPCT—3 doses	II	58	Reductions in HAMD scores from baseline at 48 hrs, 60 hrs, and day 34 of administration	Sedation and dizziness
		Dichtel et al., 2020 [58]	Pilot OL—adjunctive, uncontrolled	II	10	Total MADRS score decreased by 8 weeks, and the decrease persisted over the 2-week taper	

## 4. Glutamatergic Pharmacotherapies (Table 2)

### Ketamine and Esketamine

Recent research has increasingly focused on ketamine and its S-enantiomer esketamine as novel therapeutic interventions for preventing and treating PPD. Unlike traditional antidepressants that target monoaminergic systems, ketamine compounds work through NMDA receptor antagonism, offering rapid-onset antidepressant effects that could be particularly valuable in the peripartum period. A growing body of research has investigated various dosing regimens and administration protocols for these medications in the context of cesarean delivery and postpartum care.

A research group performed a comprehensive dose–response study with 156 participants across four groups: control (sufentanil alone) and three esketamine doses (0.1, 0.2, and 0.4 mg/kg) added to sufentanil in patient-controlled intravenous analgesia (PCIA) systems [59]. All esketamine groups demonstrated significantly reduced PPD incidence at both 1 week and 6 weeks compared to controls. The high-dose group (0.4 mg/kg) showed superior performance with the lowest effective press times (1.6 ± 0.6), minimal sufentanil consumption (60.6 ± 1.2 μg), and reduced rescue analgesia requirements (2.6% vs. 23.1% in controls, *p* < 0.004). Notably, all esketamine groups had significantly lower rates of nausea and vomiting compared to controls, likely due to reduced exposure to opioids.

The same group conducted a randomized, double-blind, controlled trial with 115 participants, without having a psychiatric diagnosis, who received either 0.2 mg/kg esketamine or saline solution intravenously after infant delivery during cesarean section under combined spinal-epidural anesthesia [60]. The study demonstrated remarkable efficacy, with PPD incidence at 1 week dropping from 15.8% in controls to 3.4% in the esketamine group (*p* < 0.01) and that at 6 weeks dropping from 19.3% to 5.2% (*p* < 0.01). EPDS scores were used with a cutoff of ≥10 for depression diagnosis. Importantly, no significant differences were observed in adverse effects between groups, including postpartum hemorrhage, nausea, vomiting, drowsiness, or nightmares, indicating a good safety profile.

A trial examined 275 puerperal women who received either standard PCIA (sufentanil 2 μg/kg + tropisetron 10 mg) or S-ketamine-enhanced PCIA (with 0.5 mg/kg S-ketamine added) [61]. The intervention provided continuous S-ketamine at approximately 0.01 mg/kg/h for 48 h. Results showed significant reductions in depression rates at 3 days (17.6% vs. 8.2%, *p* < 0.05) and 14 days (24.2% vs. 9.8%, *p* < 0.05), with corresponding decreases in EPDS scores: 7.65 ± 3.14 vs. 6.00 ± 2.47 at 3 days and 7.62 ± 3.14 vs. 6.38 ± 2.67 at 14 days (both *p* < 0.05). Additionally, Visual Analog Scale (VAS) pain scores were significantly lower 4, 8, 12, and 24 h postoperatively in the S-ketamine group.

A very comprehensive dosing study was conducted with 295 participants, examining the effects of an initial 0.25 mg/kg esketamine loading dose followed by either 1 mg/kg or 2 mg/kg esketamine PCIA [62]. Both low- and high-dose groups reported a reduced PPD symptom incidence at 7 days (29.9% placebo vs. 11.1% low dose vs. 7.1% high dose, *p* < 0.05). However, only the high-dose regimen maintained efficacy 42 days postpartum (27.8% placebo vs. 9.1% high dose, *p* < 0.05). Remission rates, defined as EPDS scores dropping from >9 to <6, were significantly higher in both esketamine groups at 7 days (35.1% placebo vs. 56.6% low dose vs. 68.7% high dose, *p* < 0.05) and 42 days. The study also demonstrated superior analgesic effects, with both esketamine groups showing significantly lower Numerical Rating Scale scores at rest within 48 h postoperatively.

A landmark randomized clinical trial conducted across five tertiary care hospitals in China demonstrated the remarkable efficacy of esketamine in treating PPD [63]. The study enrolled 364 mothers with at least mild prenatal depression (Edinburgh postnatal depression scale scores ≥ 10) and administered a single low dose (0.2 mg/kg) of esketamine intravenously over 40 min after childbirth. Results showed a dramatic reduction in major depressive episodes at 42 days postpartum, with only 6.7% of esketamine-treated mothers experiencing depression, compared to 25.4% in the placebo group. The same study demonstrated that beyond antidepressant effects, esketamine also exerted its analgesic properties, with treated mothers reporting significantly lower pain scores at rest and with movement on the first postpartum day and reduced persistent pain at 42 days (35.2% vs. 47.5%). While neuropsychiatric adverse events occurred more frequently in the esketamine group (45.1% vs. 22.0%), including dizziness, diplopia, and hallucinations, these symptoms were transient, lasting less than 24 h, and required no pharmacological intervention.

The largest individual randomized, controlled trial for the prevention of PPD in women investigated esketamine in 298 women undergoing elective cesarean delivery [64]. Participants received 0.25 mg/kg esketamine intravenously after delivery, followed by continuous administration via PCIA for 48 h. Results showed a significant reduction in depression symptom prevalence at postpartum day 7 (23.0% vs. 35.3% in controls, *p* = 0.02), with correspondingly lower EPDS scores. However, this protective effect was transient, with no significant differences observed on day 14, 28, or 42. Notably, subgroup analysis indicated that women with low baseline EPDS scores might not benefit from esketamine intervention.

A randomized, controlled trial investigated the prophylactic effects of perioperative esketamine administration on PPD risk in women undergoing elective cesarean section [65]. The study enrolled 150 participants who were randomly allocated to receive either esketamine (0.25 mg/kg intravenously during surgery followed by continuous infusion for 48 h) or placebo. The primary outcome was PPD risk assessment using the EPDS at multiple timepoints up to 6 months postpartum. Results showed that perioperative esketamine administration did not significantly reduce the incidence of PPD risk compared to the control group at any measured timepoint (3 days, 42 days, 3 months, or 6 months postpartum). However, the study did demonstrate that esketamine significantly reduced opioid consumption during the first 24 and 48 h postoperatively without increasing psychiatric adverse events.

Similar evidence is growing for the use of ketamine. An initial study investigated intraoperative ketamine administration (0.5 mg/kg) during cesarean section under general anesthesia. This initial research demonstrated that ketamine significantly reduced depression scores both two and four weeks postoperatively compared to controls, with treated patients consistently scoring below the EPDS cut-off threshold for possible PPD diagnosis [66].

Building on these previous findings, researchers conducted a larger double-blind trial involving 330 women who received either ketamine (0.25 mg/kg) or placebo within 5 min of umbilical cord clamping [67]. This study revealed a significant reduction in postpartum depressive symptoms one week postpartum (13.1% vs. 22.6%, *p* = 0.029), though notably, this protective effect did not persist at 2 weeks or 1 month. Beyond its psychiatric benefits, ketamine also provided superior postoperative analgesia, effectively reducing both wound and uterine contraction pain scores.

The importance of adequate dosing became evident in a subsequent clinical trial that demonstrated more sustained benefits when using a dose of 0.5 mg/kg [68]. In this larger study of 654 women, ketamine was administered 10 min post delivery and resulted in significantly lower rates of postpartum depression at 6–8 weeks (12.8% vs. 19.6%, *p* = 0.020) and reduced postpartum blues at 4–6 days (11.9% vs. 18.3%, *p* = 0.022). Importantly, this trial revealed that ketamine’s protective effects were most pronounced in women with moderate pregnancy stress, antenatal depressive symptoms, or antenatal suicidal ideation, suggesting that the intervention may be particularly valuable for high-risk populations.

The current evidence regarding ketamine and esketamine for PPD presents a complex but promising picture. While many randomized clinical trials have demonstrated significant reductions in PPD risk and severity, particularly in the early weeks following delivery, the benefits appear to be primarily short-term, with effects often diminishing by 42 days postpartum. Transient side effects include dizziness, hallucinations, and headache during the operative period. These findings suggest that ketamine may serve as an effective prophylactic agent against PPD, with higher doses potentially providing longer-lasting benefits, particularly in high-risk populations. Although the above-mentioned side effects occur more frequently with treatment, these adverse events are generally transient and resolve within 24 h without requiring intervention. Extended follow-up periods will be required to fully establish the clinical utility and optimal protocols for ketamine-based interventions in PPD prevention and treatment.

**Table 2:** Studies on glutamatergic compounds for the treatment of peripartum depression.

Drug	Mechanism of Action	Studies	Design	Phase	*n*	Key Findings	Side Effects
**KETAMINE**	NMDAR antagonism; AMPAR stimulation	M. Alipoor, M. Loripoor, M. Kazemi, F. Farahbakhsh, and A. Sarkoohi, 2021 [66]	RDBPCT, 2-arm	III	134	Mean EPDS score was significantly lower four weeks after the caesarian section in the ketamine group compared to placebo group.	Vomiting, headache, dizziness, hallucinations, nystagmus
		J. Yao, T. Song, Y. Zhang, N. Guo, and P. Zhao, 2020 [67]	RDBPCT, 2-arm	III	330	Significant differences were observed in the EPDS score between subjects in the ketamine group and the placebo group at 1 week postpartum. No difference was found between subjects in the two groups at 2 weeks and 1 month postpartum.	
		Ma et al., 2019 [68]	RDBPCT, 2-arm	III	654	PPD prevalence in the ketamine group was significantly lower than in the control group. The EPDS score at postpartum day 4 was significantly lower in the ketamine group compared with the control group, and the prevalence of postpartum blues was significantly lower in the ketamine group than in the control group.	
**ESKETAMINE**	NMDAR antagonism; AMPAR stimulation	W. Wang, H. Xu, B. Ling, Q. Chen, J. Lv, and W. Yu, 2022 [59]	RDBPCT, 4-arm	III	160	A lower incidence of PPD was observed at 1 week and 6 weeks in the esketamine + sufentanil group.	Nausea, vomiting, dizziness, hallucinations, somnolence, nightmares, nystagmus and postpartum hemorrhage
		W. Wang, B. Ling, Q. Chen, H. Xu, J. Lv, and W. Yu, 2023 [60]	RDBPCT, 2-arm	III	120	The incidence of PPD was significantly lower at 1 week and 6 weeks after cesarean surgery in the esketamine group.	
		Y. Han, P. Li, M. Miao, Y. Tao, X. Kang, and J. Zhang, 2022 [61]	RDBPCT, 2-arm	III	275	The rate of depression in the parturient period on postoperative days 3, 14, 28 was significantly lower in the esketamine group. EPDS scores in the esketamine group were also significantly lower on postoperative days 3,14, and 28.	
		Yang et al., 2023 [62]	RDBPCT, 3-arm	III	312	The results showed that the incidence of depression symptoms at 42 days postpartum was 27.8% for the placebo group, 14.1% for the esketamine group (1 mg/kg), and 9.1% for the esketamine group (2 mg/kg). The incidence of depression symptoms at 7 days postpartum was 29.9% in the placebo group, 11.1% for the esketamine group (1 mg/kg) and 7.1% for the esketamine group (2 mg/kg).	
		Wang et al., 2024 [63]	RDBPCT, 2-arm	III	364	EDPS scores were lower in the esketamine group at day 7. HAMD-17 scores at 42 days postpartum were also lower in the esketamine group.	
		Chen et al., 2024 [64]	RDBPCT, 2-arm	III	298	The prevalence of depression symptoms and EPDS scores were significantly lower among patients given esketamine compared with controls on postpartum day 7 but without differences between the groups at postpartum days 14, 28, and 42.	
		Liu et al., 2023 [65]	RDBPCT, 2-arm	III	150	There were no significant differences in the prevalence of PPD risk and no significant differences in EPDS scores between the two groups at 3 days, 42 days, 3 months, and 6 months postpartum.	

## 5. Conclusions and Future Directions

Significant theoretical and practical challenges remain in the emerging clinical integration of novel therapies into PPD treatment. The transition from monoaminergic theories to glutamate–GABA imbalance in the treatment for mood disorders represents a conceptual advance, yet the clinical translation needs to fill substantial gaps between mechanistic understanding and therapeutic implementation.

There are also fundamental questions about the nature of PPD itself—if it represents a discrete episode amenable to short-term intervention or a condition requiring sustained therapeutic support. Mechanistic rationales, while compelling in preclinical models, appear insufficient to predict which patients will experience lasting benefits versus those requiring repeated interventions.

Furthermore, the safety profiles of these novel agents in the peripartum period must be taken in consideration. While generally well-tolerated, the requirement for intensive monitoring and specialized administration protocols can create significant barriers to widespread implementation. This limitation is particularly concerning, given that PPD disproportionately affects populations with limited healthcare access, potentially creating a therapeutic paradox where the most innovative treatments remain inaccessible to those most in need. Another important limitation,—this time regarding future research and clinical trials—is that the emphasis on perioperative pharmacotherapeutic administration, while methodologically convenient, may not reflect the broader clinical reality of PPD. Most women developing depression during pregnancy or postpartum do not undergo cesarean delivery, raising questions about the generalizability of findings from surgical populations to the wider patient population.

Recent evidence suggests that many new pharmacotherapies for PPD targeting the GABA–glutamate pathways represent a promising new therapeutic option, with clinical applications still in early stages, following the first approval of drugs by the FDA and the possible use of available MDD pharmacotherapies. Still, the variability in dosing regimens, outcome measures, means of administration, and patient populations across studies indicates that optimal therapeutic protocols and definitive guidelines have yet to be established.

The current research reveals a critical gap between proof-of-concept efficacy and clinical implementation. Future research should focus on understanding how most efficacious pharmacotherapies work; making them more targeted; and, most importantly, creating practical delivery methods that offer real therapeutic benefits that can be used in clinical practice. The integration of these novel pharmacotherapeutic approaches represents a crucial step toward personalized peripartum psychiatric care, though considerable work remains to establish optimal patient selection criteria and treatment protocols.

Future research priorities should include the development of validated biomarkers for treatment response prediction, enabling precision medicine approaches to patient selection and dosing optimization. Comprehensive pharmacokinetic–pharmacodynamic investigations are essential to optimize dosing regimens while minimizing adverse effects.

Population-specific investigations are urgently needed, particularly examining safety and efficacy in breastfeeding women, diverse ethnic populations, and childbearing-age women with comorbid psychiatric conditions. Additionally, economic health analyses evaluating cost-effectiveness compared to conventional treatments will inform healthcare policy decisions regarding the integration of these novel therapeutic modalities into standard peripartum care protocols, making all elements of PPD standard for clinical psychiatric and obstetric care.

## Abbreviations

The following abbreviations are used in this manuscript:

RDBPCT	Randomized, Double-Blinded, Placebo-Controlled Trial
EPDS	Edinburgh Postnatal Depression Scale
PPD	Peripartum Depression
MDD	Major Depressive Disorder
NMDAR	NMDA Receptor
AMPAR	AMPA Receptor
OL	Open Label
MADRS	Montgomery–Åsberg Depression Rating Scale
HAMD	Hamilton Depression Rating Scale
RCT	Randomized Clinical Trial

## References

[1] Cimino S. (2023). Epidemiology, Etiology and Intervention Strategies for Peri-Partum Depression in Mothers. J. Clin. Med..

[2] Fox M., Sandman C.A., Davis E.P., Glynn L.M. (2018). A longitudinal study of women’s depression symptom profiles during and after the postpartum phase. Depress. Anxiety.

[3] Putnam K.T., Wilcox M., Robertson-Blackmore E., Sharkey K., Bergink V., Munk-Olsen T., Deligiannidis K.M., Payne J., Altemus M., Newport J. (2017). Clinical phenotypes of perinatal depression and time of symptom onset: Analysis of data from an international consortium. Lancet Psychiatry.

[4] Shorey S., Chee C.Y.I., Ng E.D., Chan Y.H., Tam W.W.S., Chong Y.S. (2018). Prevalence and incidence of postpartum depression among healthy mothers: A systematic review and meta-analysis. J. Psychiatr. Res..

[5] Lindahl V., Pearson J.L., Colpe L. (2005). Prevalence of suicidality during pregnancy and the postpartum. Arch. Women’s Ment. Health.

[6] Justesen K., Jourdaine D. (2023). Peripartum Depression: Detection and Treatment. Am. Fam. Physician.

[7] Langan R., Goodbred A.J. (2016). Identification and Management of Peripartum Depression. Am. Fam. Physician.

[8] Stefana A., Mirabella F., Gigantesco A., Camoni L., Aceti F., Adulti I., Aite L., Bagolan P., Barbano G., for the Perinatal Mental Health Nework (2024). The screening accuracy of the Edinburgh Postnatal Depression Scale (EPDS) to detect perinatal depression with and without the self-harm item in pregnant and postpartum women. J. Psychosom. Obstet. Gynecol..

[9] Kendall-Tackett K.A. (2024). Screening for Perinatal Depression: Barriers, Guidelines, and Measurement Scales. J. Clin. Med..

[10] Smith-Nielsen J., Egmose I., Matthey S., Stougård M., Reijman S., Væver M.S. (2025). Proposing a two-stage screening approach to distinguish between transient and enduring postnatal depressive symptoms: A prospective cohort study. Int. J. Nurs. Stud. Adv..

[11] Agrawal I., Mehendale A.M., Malhotra R. (2022). Risk Factors of Postpartum Depression. Cureus.

[12] Klainin P., Arthur D.G. (2009). Postpartum depression in Asian cultures: A literature review. Int. J. Nurs. Stud..

[13] Yang K., Wu J., Chen X. (2022). Risk factors of perinatal depression in women: A systematic review and meta-analysis. BMC Psychiatry.

[14] Gastaldon C., Solmi M., Correll C.U., Barbui C., Schoretsanitis G. (2022). Risk factors of postpartum depression and depressive symptoms: Umbrella review of current evidence from systematic reviews and meta-analyses of observational studies. Br. J. Psychiatry.

[15] Wisner K.L., Moses-Kolko E.L., Sit D.K.Y. (2010). Postpartum depression: A disorder in search of a definition. Arch. Women’s Ment. Health.

[16] Batt M.M., Duffy K.A., Novick A.M., Metcalf C.A., Epperson C.N. (2020). Is Postpartum Depression Different From Depression Occurring Outside of the Perinatal Period? A Review of the Evidence. Focus.

[17] Guintivano J., Manuck T., Meltzer-Brody S. (2018). Predictors of Postpartum Depression: A Comprehensive Review of the Last Decade of Evidence. Clin. Obstet. Gynecol..

[18] Leung B.M.Y., Letourneau N.L., Giesbrecht G.F., Ntanda H., Hart M., The APrON Team (2017). Predictors of Postpartum Depression in Partnered Mothers and Fathers from a Longitudinal Cohort. Community Ment. Health J..

[19] Silverman M.E., Reichenberg A., Savitz D.A., Cnattingius S., Lichtenstein P., Hultman C.M., Larsson H., Sandin S. (2017). The risk factors for postpartum depression: A population-based study. Depress. Anxiety.

[20] Silverman M.E., Reichenberg A., Lichtenstein P., Sandin S. (2019). Is depression more likely following childbirth? A population-based study. Arch. Women’s Ment. Health.

[21] Buttner M.M., Mott S.L., Pearlstein T., Stuart S., Zlotnick C., O’Hara M.W. (2013). Examination of premenstrual symptoms as a risk factor for depression in postpartum women. Arch. Women’s Ment. Health.

[22] Studd J., Nappi R.E. (2012). Reproductive depression. Gynecol. Endocrinol..

[23] Woody C.A., Ferrari A.J., Siskind D.J., Whiteford H.A., Harris M.G. (2017). A systematic review and meta-regression of the prevalence and incidence of perinatal depression. J. Affect. Disord..

[24] Harrington Y.A., Fortaner-Uyà L., Paolini M., Poletti S., Lorenzi C., Spadini S., Melloni E.M.T., Agnoletto E., Zanardi R., Colombo C. (2024). Disentangling the Genetic Landscape of Peripartum Depression: A Multi-Polygenic Machine Learning Approach on an Italian Sample. Genes.

[25] Cheng B., Guo Y., Chen X., Lv B., Liao Y., Qu H., Hu X., Yang H., Meng Y., Deng W. (2022). Postpartum depression and major depressive disorder: The same or not? Evidence from resting-state functional MRI. Psychoradiology.

[26] Bhatt S., Devadoss T., Manjula S.N., Rajangam J. (2021). 5-HT3 Receptor Antagonism: A Potential Therapeutic Approach for the Treatment of Depression and other Disorders. Curr. Neuropharmacol..

[27] Cipriani A., Furukawa T.A., Salanti G., Chaimani A., Atkinson L.Z., Ogawa Y., Leucht S., Ruhe H.G., Turner E.H., Higgins J.P.T. (2018). Comparative efficacy and acceptability of 21 antidepressant drugs for the acute treatment of adults with major depressive disorder: A systematic review and network meta-analysis. Lancet.

[28] Racagni G., Popoli M. (2008). Cellular and molecular mechanisms in the long-term action of antidepressants. Dialogues Clin. Neurosci..

[29] Al-Harbi K.S. (2012). Treatment-resistant depression: Therapeutic trends, challenges, and future directions. Patient Prefer. Adherence.

[30] Jiang Y., Zou D., Li Y., Gu S., Dong J., Ma X., Xu S., Wang F., Huang J.H. (2022). Monoamine Neurotransmitters Control Basic Emotions and Affect Major Depressive Disorders. Pharmaceuticals.

[31] Sanacora G., Treccani G., Popoli M. (2012). Towards a glutamate hypothesis of depression. Neuropharmacology.

[32] Albrecht J. (2007). Glutamine in the central nervous system: Function and dysfunction. Front. Biosci..

[33] Lüscher B., Möhler H. (2019). Brexanolone, a neurosteroid antidepressant, vindicates the GABAergic deficit hypothesis of depression and may foster resilience. F1000Research.

[34] Luscher B., Shen Q., Sahir N. (2011). The GABAergic deficit hypothesis of major depressive disorder. Mol. Psychiatry.

[35] MacQueen G., Frodl T. (2011). The hippocampus in major depression: Evidence for the convergence of the bench and bedside in psychiatric research?. Mol. Psychiatry.

[36] Lener M.S., Niciu M.J., Ballard E.D., Park M., Park L.T., Nugent A.C., Zarate C.A. (2017). Glutamate and Gamma-Aminobutyric Acid Systems in the Pathophysiology of Major Depression and Antidepressant Response to Ketamine. Biol. Psychiatry.

[37] Arnone D., Mumuni A.N., Jauhar S., Condon B., Cavanagh J. (2015). Indirect evidence of selective glial involvement in glutamate-based mechanisms of mood regulation in depression: Meta-analysis of absolute prefrontal neuro-metabolic concentrations. Eur. Neuropsychopharmacol..

[38] Bhagwagar Z., Wylezinska M., Jezzard P., Evans J., Boorman E., Matthews P.M., Cowen P.J. (2008). Low GABA concentrations in occipital cortex and anterior cingulate cortex in medication-free, recovered depressed patients. Int. J. Neuropsychopharmacol..

[39] Luscher B., Fuchs T. (2015). GABAergic Control of Depression-Related Brain States. Advances in Pharmacology.

[40] Koolschijn P.C.M.P., Van Haren N.E.M., Lensvelt-Mulders G.J.L.M., Hulshoff Pol H.E., Kahn R.S. (2009). Brain volume abnormalities in major depressive disorder: A meta-analysis of magnetic resonance imaging studies. Hum. Brain Mapp..

[41] Cutler A.J., Mattingly G.W., Maletic V. (2023). Understanding the mechanism of action and clinical effects of neuroactive steroids and GABAergic compounds in major depressive disorder. Transl. Psychiatry.

[42] Licheri V., Talani G., Gorule A.A., Mostallino M.C., Biggio G., Sanna E. (2015). Plasticity of GABAA Receptors during Pregnancy and Postpartum Period: From Gene to Function. Neural Plast..

[43] Deligiannidis K.M., Kroll-Desrosiers A.R., Mo S., Nguyen H.P., Svenson A., Jaitly N., Hall J.E., Barton B.A., Rothschild A.J., Shaffer S.A. (2016). Peripartum neuroactive steroid and γ-aminobutyric acid profiles in women at-risk for postpartum depression. Psychoneuroendocrinology.

[44] Payne J.L., Maguire J. (2019). Pathophysiological mechanisms implicated in postpartum depression. Front. Neuroendocrinol..

[45] Kanes S.J., Colquhoun H., Doherty J., Raines S., Hoffmann E., Rubinow D.R., Meltzer-Brody S. (2017). Open-label, proof-of-concept study of brexanolone in the treatment of severe postpartum depression. Hum. Psychopharmacol. Clin. Exp..

[46] Meltzer-Brody S., Colquhoun H., Riesenberg R., Epperson C.N., Deligiannidis K.M., Rubinow D.R., Li H., Sankoh A.J., Clemson C., Schacterle A. (2018). Brexanolone injection in post-partum depression: Two multicentre, double-blind, randomised, placebo-controlled, phase 3 trials. Lancet.

[47] Epperson C.N., Rubinow D.R., Meltzer-Brody S., Deligiannidis K.M., Riesenberg R., Krystal A.D., Bankole K., Huang M.-Y., Li H., Brown C. (2023). Effect of brexanolone on depressive symptoms, anxiety, and insomnia in women with postpartum depression: Pooled analyses from 3 double-blind, randomized, placebo-controlled clinical trials in the HUMMINGBIRD clinical program. J. Affect. Disord..

[48] Hutcherson T.C., Cieri-Hutcherson N.E., Gosciak M.F. (2020). Brexanolone for postpartum depression. Am. J. Health Syst. Pharm..

[49] Gunduz-Bruce H., Silber C., Kaul I., Rothschild A.J., Riesenberg R., Sankoh A.J., Li H., Lasser R., Zorumski C.F., Rubinow D.R. (2019). Trial of SAGE-217 in Patients with Major Depressive Disorder. N. Engl. J. Med..

[50] Kato M., Nakagome K., Baba T., Sonoyama T., Okutsu D., Yamanaka H., Shimizu R., Motomiya T., Inoue T. (2023). Efficacy and safety of zuranolone in Japanese adults with major depressive disorder: A double-blind, randomized, placebo-controlled, phase 2 clinical trial. Psychiatry Clin. Neurosci..

[51] Clayton A.H., Lasser R., Nandy I., Sankoh A.J., Jonas J., Kanes S.J. (2023). Zuranolone in Major Depressive Disorder: Results From MOUNTAIN—A Phase 3, Multicenter, Double-Blind, Randomized, Placebo-Controlled Trial. J. Clin. Psychiatry.

[52] Clayton A.H., Lasser R., Parikh S.V., Iosifescu D.V., Jung J., Kotecha M., Forrestal F., Jonas J., Kanes S.J., Doherty J. (2023). Zuranolone for the Treatment of Adults With Major Depressive Disorder: A Randomized, Placebo-Controlled Phase 3 Trial. Am. J. Psychiatry.

[53] Deligiannidis K.M., Meltzer-Brody S., Maximos B., Peeper E.Q., Freeman M., Lasser R., Bullock A., Kotecha M., Li S., Forrestal F. (2023). Zuranolone for the Treatment of Postpartum Depression. Am. J. Psychiatry.

[54] Cutler A.J., Mattingly G.W., Kornstein S.G., Aaronson S.T., Lasser R., Zhang H., Rana N., Brown C., Levin S., Miller C. (2023). Long-Term Safety and Efficacy of Initial and Repeat Treatment Courses With Zuranolone in Adult Patients With Major Depressive Disorder: Interim Results From the Open-Label, Phase 3 SHORELINE Study. J. Clin. Psychiatry.

[55] Parikh S.V., Aaronson S.T., Mathew S.J., Alva G., DeBattista C., Kanes S., Lasser R., Bullock A., Kotecha M., Jung J. (2024). Efficacy and safety of zuranolone co-initiated with an antidepressant in adults with major depressive disorder: Results from the phase 3 CORAL study. Neuropsychopharmacology.

[56] Clayton A.H., Suthoff E., Jain R., Kosinski M., Fridman M., Deligiannidis K.M., Meltzer-Brody S., Chen S.-Y., Gervitz L., Huang M.-Y. (2024). The magnitude and sustainability of treatment benefit of zuranolone on function and well-being as assessed by the SF-36 in adult patients with MDD and PPD: An integrated analysis of 4 randomized clinical trials. J. Affect. Disord..

[57] Gutierrez-Esteinou R., Maximos B., Riesenberg R., Johnson K.A., Aimetti A., Lappalainen J., Masuoka L. (2019). T136. Safety and Efficacy of Intravenous Ganaxolone in Severe Postpartum Depression: Results From a Double-Blind, Placebo-Controlled Phase 2 Study. Biol. Psychiatry.

[58] Dichtel L.E., Nyer M., Dording C., Fisher L.B., Cusin C., Shapero B.G., Pedrelli P., Kimball A.S., Rao E.M., Mischoulon D. (2020). Effects of Open-Label, Adjunctive Ganaxolone on Persistent Depression Despite Adequate Antidepressant Treatment in Postmenopausal Women: A Pilot Study. J. Clin. Psychiatry.

[59] Wang W., Xu H., Ling B., Chen Q., Lv J., Yu W. (2022). Effects of esketamine on analgesia and postpartum depression after cesarean section: A randomized, double-blinded controlled trial. Medicine.

[60] Wang W., Ling B., Chen Q., Xu H., Lv J., Yu W. (2023). Effect of pre-administration of esketamine intraoperatively on postpartum depression after cesarean section: A randomized, double-blinded controlled trial. Medicine.

[61] Han Y., Li P., Miao M., Tao Y., Kang X., Zhang J. (2022). S-ketamine as an adjuvant in patient-controlled intravenous analgesia for preventing postpartum depression: A randomized controlled trial. BMC Anesthesiol..

[62] Yang S.Q., Zhou Y.Y., Yang S.T., Mao X.Y., Chen L., Bai Z.H., Ping A.Q., Xu S.Y., Li Q.W., Gao K. (2023). Effects of different doses of esketamine intervention on postpartum depressive symptoms in cesarean section women: A randomized, double-blind, controlled clinical study. J. Affect. Disord..

[63] Wang S., Deng C.-M., Zeng Y., Chen X.-Z., Li A.-Y., Feng S.-W., Xu L.-L., Chen L., Yuan H.-M., Hu H. (2024). Efficacy of a single low dose of esketamine after childbirth for mothers with symptoms of prenatal depression: Randomised clinical trial. BMJ.

[64] Chen Y., Guo Y., Wu H., Tang Y.-J., Sooranna S.R., Zhang L., Chen T., Xie X.-Y., Qiu L.-C., Wu X.-D. (2024). Perioperative Adjunctive Esketamine for Postpartum Depression Among Women Undergoing Elective Cesarean Delivery: A Randomized Clinical Trial. JAMA Netw. Open.

[65] Liu Q.-R., Zong Q.-K., Ding L.-L., Dai H.-Y., Sun Y., Dong Y.-Y., Ren Z.-Y., Hashimoto K., Yang J.-J. (2023). Effects of perioperative use of esketamine on postpartum depression risk in patients undergoing cesarean section: A randomized controlled trial. J. Affect. Disord..

[66] Alipoor M., Loripoor M., Kazemi M., Farahbakhsh F., Sarkoohi A. (2021). The effect of ketamine on preventing postpartum depression. J. Med. Life.

[67] Yao J., Song T., Zhang Y., Guo N., Zhao P. (2020). Intraoperative ketamine for reduction in postpartum depressive symptoms after cesarean delivery: A double-blind, randomized clinical trial. Brain Behav..

[68] Ma J.-H., Wang S.-Y., Yu H.-Y., Li D.-Y., Luo S.-C., Zheng S.-S., Wan L.-F., Duan K.-M. (2019). Prophylactic use of ketamine reduces postpartum depression in Chinese women undergoing cesarean section✰. Psychiatry Res..

